# The Prognostic Value of Serum Neuron-Specific Enolase in Traumatic Brain Injury: Systematic Review and Meta-Analysis

**DOI:** 10.1371/journal.pone.0106680

**Published:** 2014-09-04

**Authors:** Feng Cheng, Qiang Yuan, Jian Yang, Wenming Wang, Hua Liu

**Affiliations:** 1 Department of Neurosurgery, The First People's Hospital of Kunshan, affiliated with Jiangsu University, Suzhou, PR China; 2 Department of Neurosurgery, Huashan Hospital, affiliated to Fudan University, Shanghai, PR China; St Michael's Hospital, University of Toronto, Canada

## Abstract

**Background:**

Several studies have suggested that neuron-specific enolase (NSE) in serum may be a biomarker of traumatic brain injury. However, whether serum NSE levels correlate with outcomes remains unclear. The purpose of this review was to evaluate the prognostic value of serum NSE protein after traumatic brain injury.

**Methods:**

PubMed and Embase were searched for relevant studies published up to October 2013. Full-text publications on the relationship of NSE to TBI were included if the studies concerned patients with closed head injury, NSE levels in serum after injury, and Glasgow Outcome Scale (GOS) or Extended GOS (GOSE) scores or mortality. Study design, inclusion criteria, assay, blood sample collection time, NSE cutoff, sensitivity and specificity of NSE for mortality prediction (if sufficient information was provided to calculate these values), and main outcomes were recorded.

**Results:**

Sixteen studies were eligible for the current meta-analysis. In the six studies comparing NSE concentrations between TBI patients who died and those who survived, NSE concentrations correlated with mortality (M.D. 0.28, 95% confidence interval (CI), 0.21 to 0.34; I^2^ 55%). In the eight studies evaluating GOS or GOSE, patients with unfavorable outcomes had significantly higher NSE concentrations than those with favorable outcomes (M.D. 0.24, 95% CI, 0.17 to 0.31; I^2^ 64%). From the studies providing sufficient data, the pooled sensitivity and specificity for mortality were 0.79 and 0.50, and 0.72 and 0.66 for unfavorable neurological prognosis, respectively. The areas under the SROC curve (AUC) of NSE concentrations were 0.73 (95% CI, 0.66–0.80) for unfavorable outcome and 0.76 (95% CI, 0.62–0.90) for mortality.

**Conclusions:**

Mortality and unfavorable outcome were significantly associated with greater NSE concentrations. In addition, NSE has moderate discriminatory ability to predict mortality and neurological outcome in TBI patients. The optimal discrimination cutoff values and optimal sampling time remain uncertain because of significant variations between studies.

## Introduction

Traumatic brain injury (TBI) is a common public health and socio-economic problem worldwide. TBI is a major cause of death and lifelong disability, especially among young adults [1]. Despite recent improvements in management of TBI in intensive care and the development of standardized guidelines, mortality and morbidity in these patients remain high [2]. Early determination of prognosis based on epidemiological data is key to inform care of these patients [3], but current prognostic models based on demographics, clinical examination, and radiological imaging have limited predictive capacity [4]. Thus, other prognostic indicators may be more useful for early prediction of outcomes in TBI patients [5].

Over the past several years, biomarkers of brain injury have been increasingly investigated as potential tools for prognostic evaluation [6]–[7]. Neuron-specific enolase (NSE), first described by Moore and McGregor in 1965[8], is a 78-kDa dimeric γ-isoenzyme of the glycolytic enzyme enolase, localized predominately in the cytoplasm of neurons, which participates in slow axoplasmic transport [9]–[10]. NSE is not normally secreted, but when axons are damaged, NSE is upregulated to maintain homeostasis [11]. Therefore, NSE is the only marker that directly assesses functional damage to neurons. For many TBI patients, especially the severe TBI, NSE value keeps high or increases secondarily and leads to a second peak value due to the secondary brain injury. In addition, in patients with widespread brain lesions and more and more serious secondary brain injury, NSE values are persistently elevated. Therefore, the NSE levels not only can reflect the extent of primary brain damage, but also reflect the progression of secondary damage. Accordingly, NSE has excellent theoretical potential as a long-term prognostic biomarker and therapeutic indicator in neurological intensive care [12]–[13]. Several studies have suggested increased NSE concentrations in blood following TBI, indicating a potential clinical role as a biomarker of this injury [14]–[17]. However, its association with outcome remains unclear. NSE is not often measured in clinical practice because it is not considered a standard indicator. Thus, we conducted a meta-analysis to evaluate the prognostic value of serum NSE concentrations after traumatic brain injury.

## Methods

### Data Sources and Search Strategy

Two investigators searched MEDLINE and EMBASE for relevant articles published up to October 2013 for the following Medical Headings and text words: “NSE,” “neuron specific enolase,” “craniocerebral trauma,” “closed head trauma,” “brain injuries,” “traumatic brain injury,” and related terms. The syntax for the MEDLINE searches was as follows: (NSE[Text Word]) OR (neuron specific enolase[Text Word]) AND ((“brain injuries”[MeSH Terms]) OR (“craniocerebral trauma”[MeSH Terms]) OR (“brain hemorrhage, traumatic”[MeSH Terms]) OR (“brain stem hemorrhage, traumatic”[MeSH Terms]) OR (“subarachnoid hemorrhage, traumatic”[MeSH Terms]) OR (closed head injury[Text Word]) OR (closed head trauma[Text Word]) OR (traumatic brain injury[Text Word]) OR (brain injury[Text Word]) OR (TBI[Text Word]) OR (CHI[Text Word])). We performed this relatively wide search to include the maximum number of relevant patients.

### Study Selection

The reference lists of the included studies and review articles were also checked manually for further eligible studies. Full-text publications concerning the relationship of NSE to TBI outcomes were included if the studies contained patients with closed head injury, NSE levels in serum after injury, and GOS or GOSE scores or mortality. Included studies had to report both at least one outcome of interest and NSE concentrations in venous blood or arterial blood, measured quantitatively in the emergency room or the intensive care unit. Searches were restricted to English language literature on human subjects only. Abstracts or meeting proceedings were excluded.

### Data Abstraction

Data were extracted independently by two reviewers and any uncertainties or disagreements were resolved by discussion. The following were recorded or calculated: first author, year of publication, study design, age of patients, inclusion criteria, assay, blood sample collection time, cutoff NSE value to distinguish between outcomes, sensitivity and specificity of prediction (if sufficient data were available), main outcome, and relevant results with respect to the key question, including predictive statistics. If certain key factors or data were missing, authors were contacted for clarification. In the case of multiple studies from the same research group, authors were also contacted to ensure unique patients. Because a cutoff of 20 µg/L has been reported independently by several research groups, results in relation to this level were extracted, if possible, to attempt an interpretation of data using the same cutoff.

### Risk of Bias of Included Studies

The quality of the selected studies was assessed as recommended in the Standards for the Reporting of Diagnostic Accuracy (STARD) by using QUADAS-2 assessment tool [18].

### Statistical Analysis

Statistical analyses were performed using Review Manager version 5.2 (Cochrane Collaboration, Oxford, UK), STATA version 10 (Stata Corp., College Station, TX) and Meta-DiSc version 1.4. All statistical tests were two sided, with P values less than 0.05 demonstrating statistical significance. Mean differences (M.D.s) were used for the analysis of continuous variables (NSE concentrations). The distribution of NSE concentrations was right-skewed and we thus log-transformed them to yield a normal distribution, facilitating analysis and distinction of outcomes between groups [19]. Therefore, a mean difference greater than 0 indicates that mean concentrations are higher in the group with unfavorable outcomes. The I-squared (I^2^) statistic was used to measure the extent of inconsistency among the results [20]. Heterogeneity was detected using the chi-squared (χ^2^) test. Because the χ^2^ test lacks power when the number of studies is small, we considered significant heterogeneity to be present when both the χ^2^ value was within the 10% level of significance (P<0.10) and the I^2^ value exceeded 50%. In cases of heterogeneity, summary measures of the effect of NSE on mortality or neurological outcome were obtained by conducting a random-effects meta-analysis of the best-effect estimate available from each study, which assumes that studies were a random sample of a hypothetical population of studies and assigns a weight to each study, taking into account variance within and between studies. Publication bias was assessed using the “funnel plot” technique, based on a graph plotting effect estimates against sample size.

By undertaking a bivariate regression approach, we calculated the pooled estimates of sensitivity (SEN) and specificity (SPE) for NSE as a predictor of mortality and GOS, and constructed hierarchical summary receiver operating characteristic (HSROC) curves [21]. Based on random-effects models, this bivariate approach investigates potential between-study heterogeneity and incorporates the possible correlation between SEN and SPE. Using the pooled SEN and SPE, positive and negative likelihood ratios (PLR and NLR, respectively) were also calculated. Heterogeneity was assessed by testing the inconsistency (I^2^) of the pooled SEN and SPE. We also computed discrimination threshold values for 100% specificity or 100% sensitivity for each of these studies.

## Results

### Study Selection

**Figure 1:**
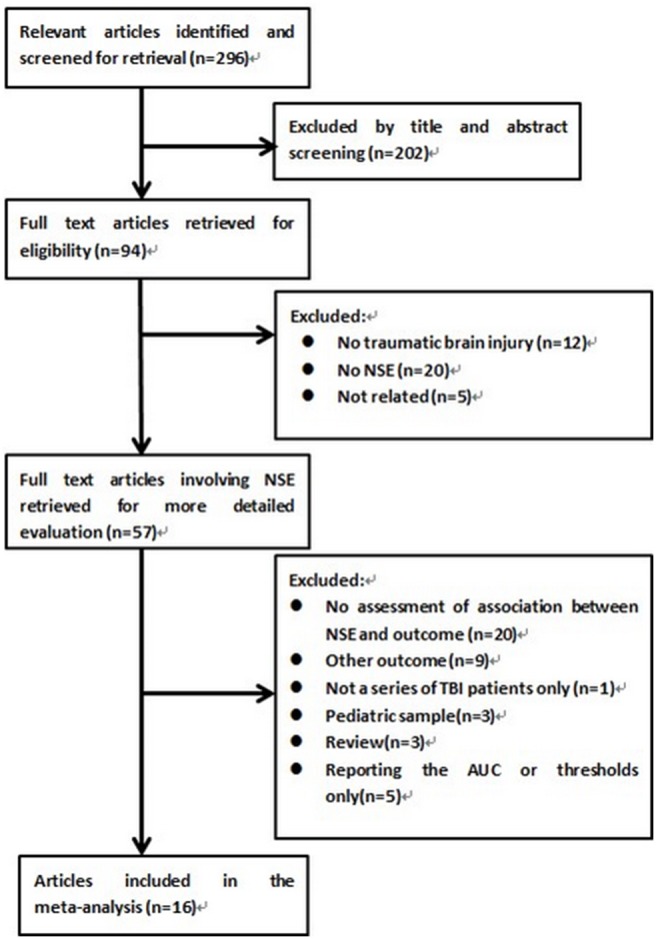
Identification process for eligible studies. Of the 296 studies initially identified from our electronic search, 16 met the inclusion criteria and were included in this meta-analysis.

In total, 296 studies were examined and screened for retrieval using the strategy described above. After screening the title and abstract, 202 studies were excluded and 94 full manuscripts were investigated in detail. Based on the inclusion and exclusion criteria, 78 of those studies were excluded, and so 16 studies were eligible for the current review and meta-analysis, consisting of 711 patients (Fig. 1). The characteristics of these studies are presented in Table 1. All included studies were published, peer-reviewed papers.

**Table 1:** Characteristics of the studies included in the meta-analysis of the prognostic value of NSE in patients with traumatic brain injury.

Author,Number ofPatients	PublishedDate	StudyDesign	Inclusion criteria	Age(years)*	Assay	Blood samplecollection time	Sensitivity and Specificity	NSE cutoff	Main outcome
**Vos et al [27]** **,** **n = 78**	2004	NR	GCS ≤8 after resuscitation.Admitted within 36 h afterinjury.	32 (15–81)	LIA	Hospital admission	Reported	21.7 µg/L	Mortality and GOS at 6 months: 1 deceased, 1–3 unfavourable, 4–5 favourable
**Li et al [26]** **,** **n = 40**	2004	Prospectivecohort	GCS ≤8. No severe systemicinjury. No heart or renalfailure. No severe infectionof central nervous system	NR	RIA	12 hours after injury	Reported	20/30 µg/L	GOS at 6 months: 1–3 unfavourable, 4–5 favourable
**Raabe et al** **[24]** **, n = 82**	1999	Prospectivecohort	GCS ≤8 after resuscitation.Admitted to neurosurgical ICU	38(16–85)	RIA	Hospital admissionand every 24 h fora maximum of 10consecutive days	Reported	20/30/100 µg/L	GOS at 6 months: 1–3 unfavourable, 4–5 favourable
**Stein et al** [**30**] **, n = 24**	2012	Prospectivecohort	Age >17. Admission withinfirst 6 h after injury. GCSscore <9 on admission.Placement of clinicallyindicated ICP monitor	30.7±12.3	ELISA	Hospital admissionand twice daily atstandard times for7 days	Not reported	NR	GOSE at 3 months, 6 months and 1 year: 1–4 unfavourable, 5–8 favourable
**Woertgen et al** **[25]** **, n = 30**	1997	Prospectivecohor	GCS ≤8. Admitted between1–6 h after injury. No spinalcord injury. No history ofneurological disease. Noresuscitation or shock	32(17–73)	RIA	Hospital admission(mean 2.5 hours), 6,12, and 24 hours aftertrauma and every 24hours up to the fifth dayafter injury.	Not reported	NR	GOS at discharge: 1–2 unfavourable,3–5 favourable
**Dauberschmidt** **et al ** **[32]** **, n = 9**	1983	NR	Cerebral coma (GlasgowComa Scale: 4 points) aftersevere head trauma	20–69	RIA	initial value	Reported	20 µg/L	Mortality at discharge
**Baker et al** **[28]** **, n = 64**	2009	Randomizedcontrolled trial	Glasgow Coma Scale (GCS)score≤8. No primary penetratinginjury, previous intravenoustherapy <50 mL, a time intervalbetween arrival at scene andintravenous access≤4 h,age≥16 years	41.4±18.8(18.3–87.9)	ELISA	ED admission, andsubsequently at 12, 24,and 48 hpost-resuscitation	Not reported	NR	Mortality at discharge
**Meric et al ** **[35]** **, n = 20**	2010	Prospectivecohort	Glasgow Coma Scale (GCS)score≤8	31(18–88)	ECLIA	Admission	Reported	20 µg/L	Mortality and GOS at 30 days: 1 deceased, 1–3 unfavourable, 4–5 favourable
**Guan et al ** **[37]** **, n = 41**	2003	Prospectivecohort	Glasgow Coma Scale (GCS)score≤8.admission within 6 hoursafter injury; closed TBI, nohistory of disease of vital organssuch as heart, brain, and kidney.	44(5–92)	ELISA	Hospital admission andevery 24 hours thereafterfor a maximum of 10consecutive days	Reported	60 µg/L	Mortality and GOS at 6 months: 1 deceased, 1–3unfavourable, 4–5 favourable
**Olivecrona et al ** **[29]** **, n = 48**	2009	Prospectivecohort	Verified head injury, GCS (8 atthe time of sedation and intubation, age between 15 and 70 years, initialcerebral perfusion pressure (CPP)of >10 mm Hg and arrival in ourdepartment within 24 h of the trauma.	30.5 (15–63)	LIAISON	Hospital admission	Reported	9.52 µg/L/11.62 µg/L	Mortality and GOS at 3 Months and 12 months: 1 deceased, 1–3 unfavourable, 4–5 favourable
**McKeating et al** **[31]** **, n = 21**	1998	NR	GCS 3–13	35(17–69)	RIA	On admission, at 24 hours,48 hours and 96 hours	Reported	20 µg/L	GOS at 6 Months: 1–3 unfavourable, 4–5 favourable
**Gradisek et al** **[33]** **, n = 84**	2012	NR	Glasgow Coma Scale≤12 afterresuscitation and was verified byCT within 2 hours of injury. Onlythe patients with GCS≤8 (n = 65, 77%)after resuscitation in the emergencyroom (ER) or those whose GCSdeteriorated to≤8 within 24 hoursafter hospital admission (n = 19, 23%)were included in the study.	46 (15–87)	LIA	At admission in the ERand then 6, 12, 24, 48,72and 96 hours post-injury	Not reported	NR	Mortality and GOS at 12 months: 1 deceased, 1–3 unfavourable, 4–5 favourable
**Ross et al ** **[17]** **, n = 9**	1996	NR	Admission within 24 h of severehead injury	21.5(16–38.5)	RIA	Initial sample	Reported	20 µg/L	GOS at discharge: 1–3 unfavourable, 4–5 favourable
**Sogut et al ** **[36]** **, n = 100**	2010	Prospectivecohort	Head trauma as the primary injury;a history of posttraumatic loss ofconsciousness or amnesia; consentprovided by the patient or their legalguardian.	18.05±13.83	ECLIA	Within 12 h after thehead injury	Not reported	NR	Mortality in the early posttraumaticperiod
**Yamazaki et al** **[34]** **, n = 17**	1995	NR	GCS 3–15	45(14–91)	RIA	1.5–8.0 hours after injury	Reported	20 µg/L	Mortality at discharge
**Raabe et al** **[23]** **, n = 44**	1998	Prospectivecohort	Glasgow Coma Scale (GCS)score≤8	41(16–83)	RIA	6±24 (median 12 hours)after admission	Not reported	NR	GOS at 6 Months: 1–3 unfavourable, 4–5 favourable

ECLIA = electrochemiluminescence immunoassay; ELISA = enzyme linked immunosorbant assay; LIA = luminescence immunoassay; RIA = radioimmunoassay; GCS = Glasgow coma scale; GOS = Glasgow outcome scale; GOSE = extended Glasgow outcome scale; NR = not reported. *Median (range) or Mean ± standard deviation.

### Study Characteristics

Fifteen studies were observational and one was a randomized controlled trial. The number of TBI patients included in the cohorts ranged from 9 to 100. Only one study reported the proportion of patients with isolated TBI, which represented 9% of its sample. The main outcome measures presented were the Glasgow outcome score (GOS) (11 studies), Glasgow outcome score extended (GOSE) (one study), and mortality (9 studies). The time of evaluation of main outcome was at discharge (six studies), 1 month (one study), 3 months (one study), 6 months (six studies), and 12 months after injury (three studies); five studies presented data from multiple time points after injury and eleven studies presented individual patient data. The site of sampling for measurement of serum NSE concentrations was venous (15 studies) or arterial (1 study). Eight studies used the radioimmunoassay (RIA) (total of 252 patients), two used a luminescent immunoassay (LIA) (162 patients), three used enzyme-linked immunosorbent assays (ELISA) (129 patients), two used electrochemiluminescent immunoassays (ECLIA) (120 patients), and the final study used a LIAISON assay (48 patients). Two studies included patients with mild TBI, two included cases of moderate TBI, and twelve included only severe TBI. Fourteen studies presented the initial NSE concentration of serial samples, five presented the peak concentration, and one presented the mean concentration. In 10 of the studies, data concerning a cutoff value of NSE concentration allowed analysis of the discriminative capacity of NSE concentration to predict mortality and neurological outcome. In addition, 12 studies presented the median and interquartile range of NSE concentrations, enabling analysis of the pooled value.

### Methodological Quality of Included Studies

**Figure 2:**
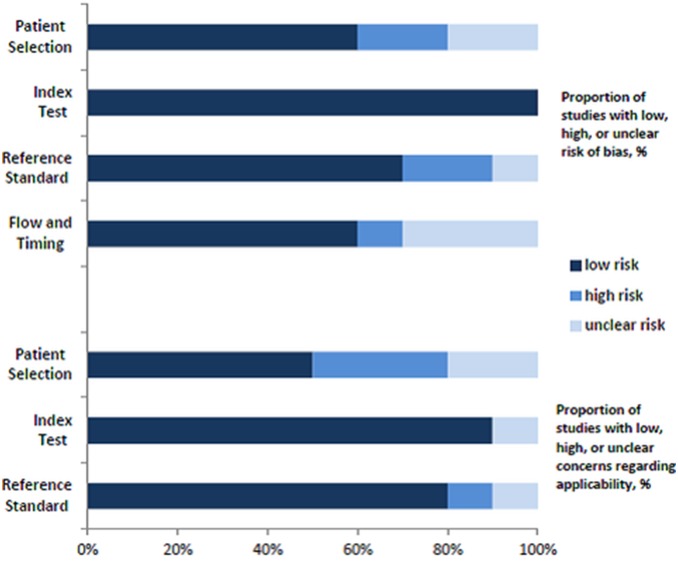
Risk of bias and applicability concerns of included studies examining role of NSE concentrations in prognosis in patients with traumatic brain injury.

Sixteen studies examined risk of bias and applicability using a modified QUADAS-2 assessment. Figure 2 presents a more complete evaluation of the methodological quality and risk of bias. Outcome assessment was blinded from NSE concentrations in 4 studies.

### Data Synthesis and Meta-Analysis

**Figure 3:**
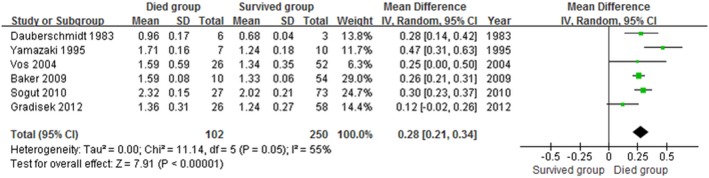
Association between NSE (shown as mean (SD) in transformed concentration) and mortality in patients with traumatic brain injury.

**Figure 4:**
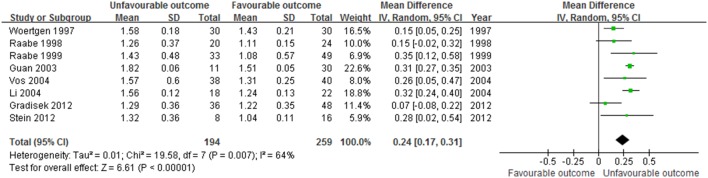
Association between NSE (shown as mean (SD) in transformed concentration) and unfavourable outcome in patients with traumatic brain injury.

**Figure 5:**
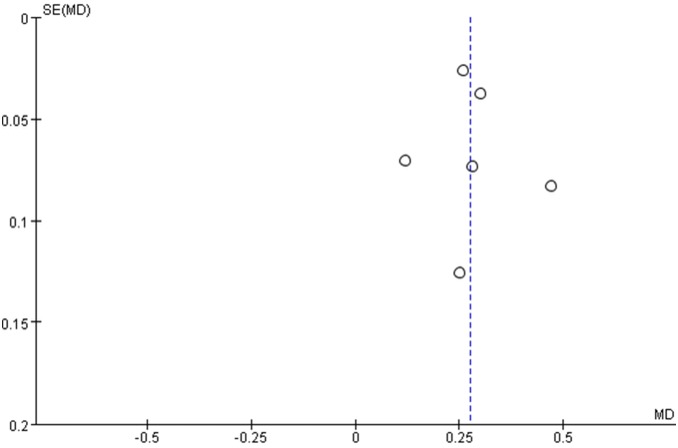
Funnel plot for the studies included to analysis the association between NSE concentration and mortality in patients with traumatic brain injury.

**Figure 6:**
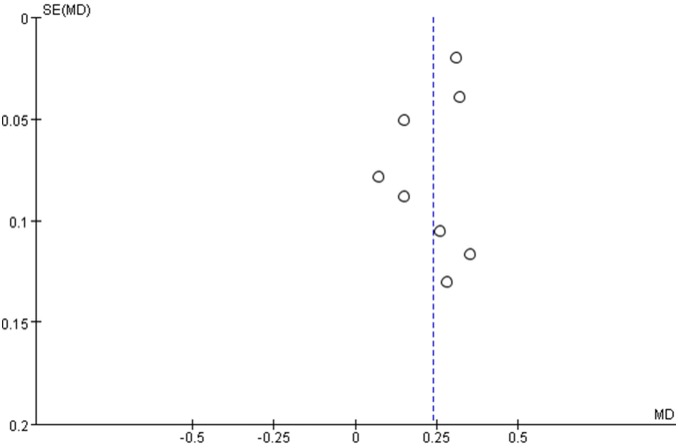
Funnel plot for the studies included to analysis the association between NSE concentration and unfavourable outcome in patients with traumatic brain injury.

The meta-analysis revealed significant positive associations between serum concentrations of NSE and outcome. Six studies compared NSE concentrations between patients who died and those who survived; NSE concentrations were significantly higher among patients who died (M.D. 0.28, 95% confidence interval 0.21 to 0.34; I^2^ 55%; Fig. 3). In the eight studies that evaluated GOS or GOSE, patients with unfavorable outcomes had significantly higher NSE concentrations than those with favorable outcomes (M.D. 0.24, 95% confidence interval 0.17 to 0.31; I^2^ 64%; Fig. 4). The results were consistent in all sensitivity analyses. In mortality and outcome subgroup analyses, heterogeneity was lower among patients evaluated at the same time post-injury (Tables 2 and 3). Analysis of outcome subgroups also revealed lower heterogeneity among patients with minimally severe injury (Table 3). No publication bias was detected by the funnel plots (Figs. 5 and 6).

**Table 2:** Sensitivity analyses for association of NSE concentrations with mortality in patients with traumatic brain injury.

	No of studies	MD (95% Cl)	I^2^ (%)
**Minimal severity of traumatic brain injury:**			
** Mild**	**2**	**0.37 [0.21, 0.53]**	**71**
** Moderate**	**1**	**0.12 [-0.02, 0.26]**	**–**
** Severe**	**3**	**0.26 [0.21, 0.31]**	**0**
**Assay:**			
** ELISA**	**1**	**0.26 [0.21, 0.31]**	**–**
** RIA**	**2**	**0.37 [0.18, 0.56]**	**66**
** LIA**	**2**	**0.15 [0.03, 0.27]**	**0**
** ECLIA**	**1**	**0.30 [0.23, 0.37]**	**–**
**Prognostic evaluation time:**			
** At discharge**	**4**	**0.30 [0.24, 0.37]**	**50**
** 6 months**	**1**	**0.25 [0.00, 0.50]**	**–**
** 12 months**	**1**	**0.12 [-0.02, 0.26]**	**–**
**Blood sample type:**			
** Arterial**	**0**	**–**	**–**
** Venous**	**6**	**0.28 [0.21, 0.34]**	**55**
**Concentration measurement type:**			
** Initial concentrations**	**5**	**0.28 [0.18, 0.39]**	**63**
** Peak concentrations**	**2**	**0.26 [0.21, 0.31]**	**0**
** Mean daily concentrations**	**0**	**–**	**–**
**Isolated traumatic brain injury:**			
** Isolated**	**1**	**0.26 [0.21, 0.31]**	**–**
** Multiple trauma or unspecified**	**5**	**0.28 [0.18, 0.39]**	**63**

**ECLIA = electrochemiluminescence immunoassay; ELISA = enzyme linked immunosorbant assay; LIA = luminescence immunoassay; RIA = radioimmunoassay.**

**Table 3:** Sensitivity analyses for association of NSE concentrations with unfavourable outcome in patients with traumatic brain injury.

	No of studies	MD (95% Cl)	I^2^ (%)
**Minimal severity of traumatic brain injury:**			
** Mild**	**0**	**–**	**–**
** Moderate**	**1**	**0.07 [-0.08, 0.22]**	**–**
** Severe**	**7**	**0.26 [0.20, 0.33]**	**50**
**Assay:**			
** ELISA**	**2**	**0.31 [0.27, 0.35]**	**0**
** RIA**	**4**	**0.24 [0.13, 0.35]**	**67**
** LIA**	**2**	**0.15 [-0.03, 0.34]**	**52**
** ECLIA**	**0**	**–**	**–**
**Prognostic evaluation time:**			
** At discharge**	**1**	**0.15 [0.05, 0.25]**	**–**
** 6 months**	**5**	**0.31 [0.27, 0.34]**	**0**
** 12 months**	**2**	**0.15 [-0.05, 0.35]**	**48**
**Sample type:**			
** Arterial**	**1**	**0.28 [0.02, 0.54]**	**–**
** Venous**	**7**	**0.24 [0.16, 0.31]**	**69**
**Concentration measurement type:**			
** Initial concentrations**	**7**	**0.23 [0.15, 0.31]**	**69**
** Peak concentrations**	**4**	**0.30 [0.13, 0.47]**	**86**
** Mean daily concentrations**	**1**	**0.09 [-0.16, 0.34]**	**–**
**Isolated traumatic brain injury:**			
** Isolated**	**0**	**–**	**–**
** Multiple trauma or unspecified**	**8**	**0.24 [0.17, 0.31]**	**64**

**ECLIA = electrochemiluminescence immunoassay; ELISA = enzyme linked immunosorbant assay; LIA = luminescence immunoassay; RIA = radioimmunoassay.**

**Figure 7:**
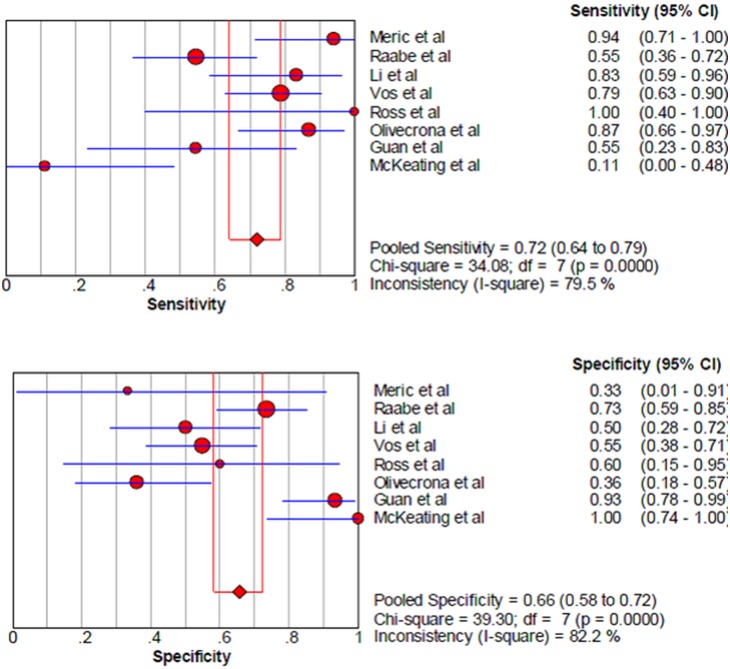
The pooled sensitivity and specificity to predict unfavourable outcome in patients with traumatic brain injury.

**Figure 8:**
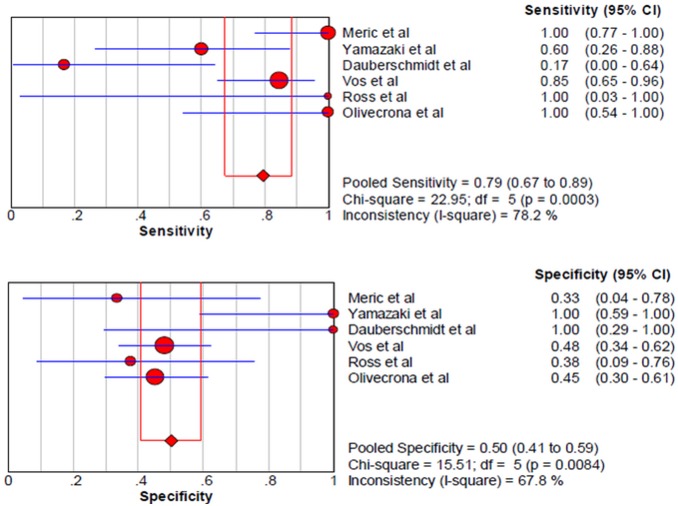
The pooled sensitivity and specificity to predict mortality in patients with traumatic brain injury.

**Figure 9:**
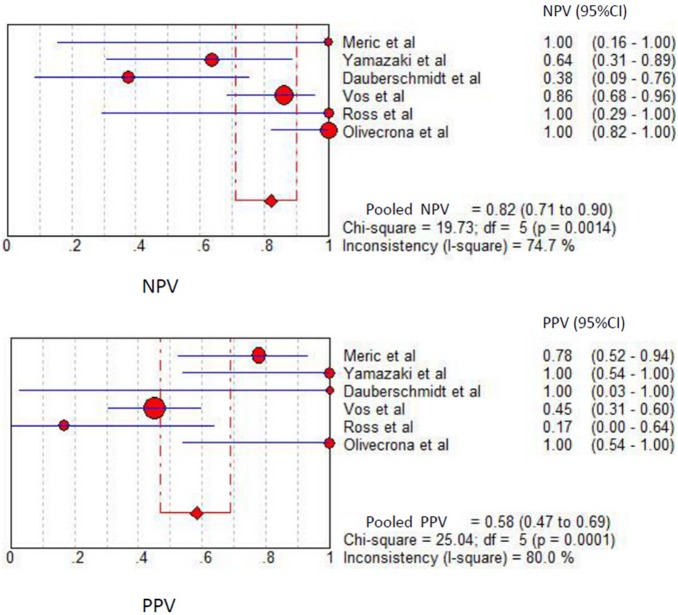
The pooled positive predictive value (PPV) and negative predictive value (NPV) to predict mortality in patients with traumatic brain injury.

**Figure 10:**
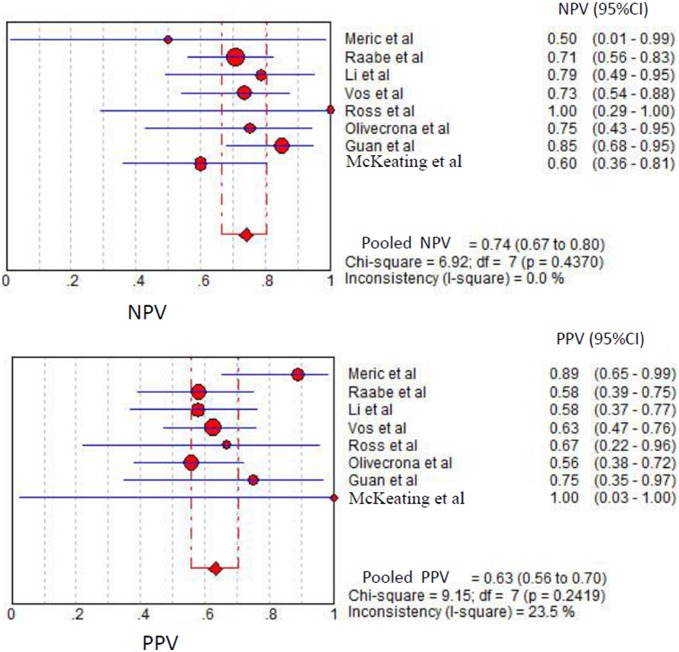
The pooled positive predictive value (PPV) and negative predictive value (NPV) to predict unfavourable outcome in patients with traumatic brain injury.

**Figure 11:**
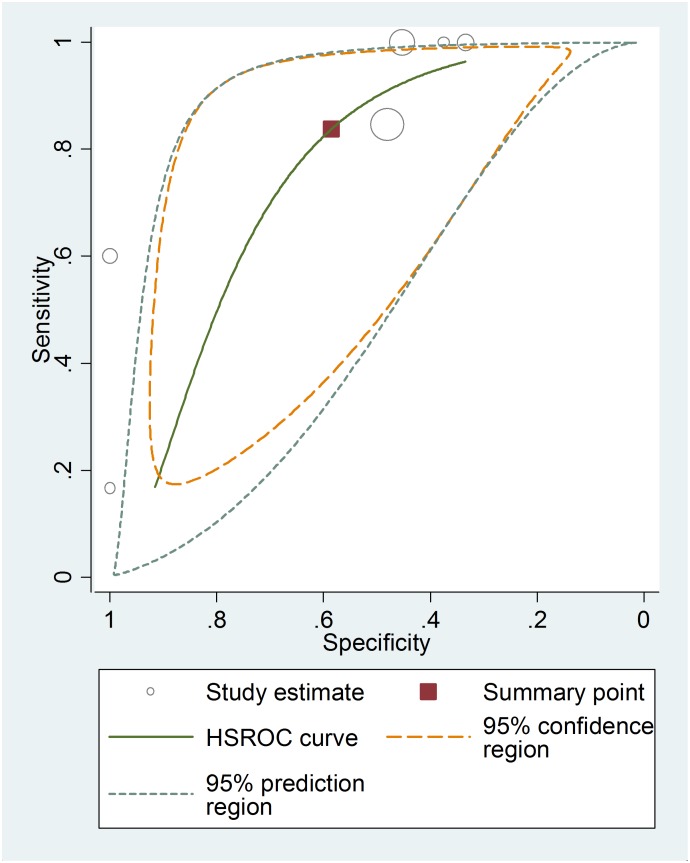
The hierarchical summary receiver operating characteristic (HSROC) curves of NSE to predict mortality in patients with traumatic brain injury.

**Figure 12:**
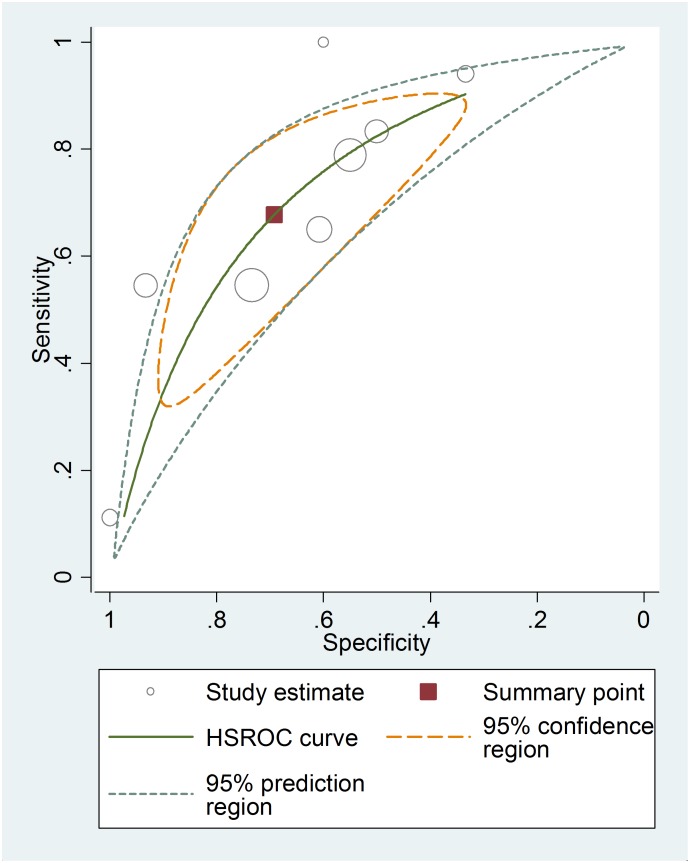
The hierarchical summary receiver operating characteristic (HSROC) curves of NSE to predict unfavourable outcome in patients with traumatic brain injury.

Among the studies presenting sufficient data for calculation, the pooled sensitivity and specificity for unfavorable neurological prognosis (GOS ≤3 or GOSE ≤4) was 0.72 (95% CI 0.64–0.79) and 0.66 (95% CI 0.58–0.72), respectively (eight studies) (Fig. 7). The pooled sensitivity and specificity for mortality was 0.79 (95% confidence interval (CI) 0.67–0.89) and 0.50 (95% CI 0.41–0.59), respectively (six studies) (Fig. 8). The results were consistent in all subgroup analyses. Heterogeneity was not influenced by any of these covariates (Tables 4 and 5). All I^2^ values were above 50%, indicating substantial heterogeneity among studies for all modalities. In addition, the pooled positive predictive value (PPV) and negative predictive value (NPV) for mortality was 0.58 (95% confidence interval (CI) 0.47–0.69) and 0.82 (95% CI 0.71–0.90), respectively (Fig. 9). The pooled PPV and NPV for unfavorable neurological prognosis was 0.63 (95% CI 0.56–0.70) and 0.74 (95% CI 0.67–0.80), respectively (Fig. 10). The HSROC curve represents the relationship between specificity and sensitivity across studies, revealing any threshold effects. Based on the bivariate approach, which estimates not only the strength but also the shape of the correlation between specificity and sensitivity, a 95% confidence ellipse and a 95% prediction ellipse were drawn (Figs. 11 and 12). The area under the SROC curve (AUC) was 0.73 (95% CI 0.66–0.80) for unfavorable outcome and 0.76 (95% CI 0.62–0.90) for mortality, signifying a moderate discriminatory ability of NSE concentrations.

**Table 4:** Sensitivity analyses for the sensitivity and specificity of NSE concentrations to predict the mortality in patients with traumatic brain injury.

	No of studies	Sensitivity	I^2^ (%)	Specificity	I^2^ (%)
**Minimal severity of traumatic** **brain injury:**					
** Mild**	**1**	**0.6**	**–**	**1**	**–**
** Moderate**	**0**	**–**	**–**	**–**	**–**
** Severe**	**5**	**0.830(0.702–0.919)**	**80.5**	**0.468(0.373–0.566)**	**25.4**
**Assay:**					
** Liason**	**1**	**1**	**–**	**0.452**	**–**
** RIA**	**3**	**0.471(0.230–0.722)**	**56.9**	**0.722(0.465–0.903)**	**81.3**
** LIA**	**1**	**0.846**	**–**	**0.481**	**–**
** ECLIA**	**1**	**1**	**–**	**0.333**	**–**
**Prognostic evaluation time:**					
** At discharge**	**3**	**0.471(0.230–0.722)**	**56.9**	**0.722(0.465–0.903)**	**81.3**
** 1 month**	**1**	**1**	**–**	**0.333**	**–**
** 3 months**	**1**	**1**	**–**	**0.452**	**–**
** 6 months**	**1**	**0.846**	**–**	**0.481**	**–**
** 12 months**	**1**	**1**	**–**	**0.45**	**–**
**Cutoff:**					
**20** **µg/L**	**4**	**0.710(0.520–0.858)**	**83.8**	**0.625(0.406–0.812)**	**77.8**
** Others**	**2**	**0.875(0.710–0.965)**	**44.1**	**0.468(0.364–0.574)**	**0**

**ECLIA = electrochemiluminescence immunoassay; ELISA = enzyme linked immunosorbant assay; LIA = luminescence immunoassay; RIA = radioimmunoassay.**

**Table 5:** Sensitivity analyses for the sensitivity and specificity of NSE concentrations to predict unfavourable outcome in patients with traumatic brain injury.

	No of studies	Sensitivity	I^2^ (%)	Specificity	I^2^ (%)
**Minimal severity of traumatic** **brain injury:**					
** Mild**	**0**	**–**	**–**	**–**	**–**
** Moderate**	**1**	**0.111**	**–**	**1**	**–**
** Severe**	**7**	**0.757(0.679–0.824)**	**67.3**	**0.632(0.556–0.704)**	**79.1**
**Assay:**					
** Liason**	**1**	**0.870**	**–**	**0.360**	**–**
** RIA**	**4**	**0.594(0.464–0.715)**	**83.8**	**0.705(0.598–0.797)**	**76.7**
** LIA**	**1**	**0.789**	**–**	**0.550**	**–**
** ECLIA**	**1**	**0.941**	**–**	**0.333**	**–**
** ELISA**	**1**	**0.545**	**–**	**0.933**	**–**
**Prognostic evaluation time:**					
** At discharge**	**1**	**1**	**–**	**0.6**	**–**
** 1 month**	**1**	**0.941**	**–**	**0.333**	**–**
** 3 months**	**1**	**0.870**	**–**	**0.360**	**–**
** 6 months**	**5**	**0.642(0.545–0.732)**	**79.9**	**0.712(0.634–0.783)**	**85**
** 12 months**	**1**	**0.650**	**–**	**0.607**	**–**
**Concentration measurement type:**					
** Initial concentrations**	**7**	**0.767(0.681–0.839)**	**78.7**	**0.628(0.541–0.709)**	**84**
** Peak concentrations**	**1**	**0.545**	**–**	**0.735**	**–**
**Cutoff:**					
**20** **µg/L**	**5**	**0.667(0.553–0.768)**	**85.5**	**0.692(0.587–0.785)**	**72.6**
** Others**	**3**	**0.778(0.664–0.867)**	**52.3**	**0.621(0.516–0.719)**	**91.5**

**ECLIA = electrochemiluminescence immunoassay; ELISA = enzyme linked immunosorbant assay; LIA = luminescence immunoassay; RIA = radioimmunoassay.**

Considering the five studies in which a cutoff of 20 µg/L for unfavorable neurological prognosis could be evaluated, pooled sensitivity and specificity were 0.67 (95% CI 0.55–0.77) and 0.69 (95% CI 0.59–0.79), respectively. Among the four studies allowing evaluation of the NSE cutoff value of 20 µg/L for mortality, the pooled sensitivity and specificity were 0.71 (95% CI 0.52–0.86) and 0.63 (95% CI 0.41–0.81), respectively. When each study was considered individually, the respective serum thresholds to attain 100% sensitivity for prognosis of death, meaning that all mortality is correctly predicted with no false negatives, ranged from 11.62 to 20 µg/L, with an associated specificity ranging from 0.33 to 0.45. In contrast, the serum threshold to attain 100% sensitivity for prognosis of unfavorable outcome was 20 µg/L, with an associated specificity of 60%.

## Discussion

This meta-analysis found a significant association between NSE serum concentration and mortality and neurological outcome in TBI patients. Mortality was associated with significantly higher NSE concentrations (M.D. 0.28, 95% confidence interval 0.21 to 0.34; I^2^ 55%), as was unfavorable outcome (M.D. 0.24, 95% confidence interval 0.17 to 0.31; I^2^ 64%). The serum values associated with 100% sensitivity for mortality and unfavorable outcome were a range of 11.62 to 20 µg/L and 20 µg/L, respectively. Our findings are highly relevant to the prognosis of TBI patients in critical condition.

There are several limitations to our meta-analysis. The major limitation was the relative dearth of studies that met our inclusion criteria. Second, there was considerable heterogeneity for all outcomes of interest. Nonetheless, sensitivity analyses did not identify a variable mediating heterogeneity in Glasgow outcome score and mortality, though many variables, including prognostic evaluation time after injury, severity of injury, measurement type, sampling type, and isolated versus multiple trauma, were assessed. Third, the time of NSE concentration measurement could be a confounding factor. Among studies where more than one sample was collected, NSE concentrations between 12 and 24 h after admission showed a stronger association with outcomes, which could reflect the impact of secondary neurological injuries such as hypotension, hypoxemia, and intracranial hypertension. Fourth, though we carried out our meta-analysis according to high methodological standards [22], the results of the meta-analysis are limited by the quality of studies included. For example, only four studies reported an outcome assessment that was blinded from NSE concentrations, which implies a high risk of bias. Moreover, we cannot exclude potential publication bias. Fifth, we could not perform sensitivity analyses related to age, pupillary reactivity, or the motor component of the Glasgow outcome score, which are known indicators of prognosis, because of variable presentation or absence of these data in included studies. Sixth, the type of NSE assay could have affected the accuracy and precision of the threshold NSE concentrations. Although our sensitivity analyses did not reveal any major impact on the results, some assays were used in only a few studies, thus precluding a robust interpretation of their impact.

Finally, NSE normally increases in the first 12 h after trauma and decreases within hours or days; its half-life is ∼24 h. Secondary increases may occur in patients with fatal outcomes. Although NSE initially appeared to be a promising marker of injury severity owing to a number of theoretical advantages, including its correlation with the number of affected neurons rather than glial cells and its high specificity for the brain [38], like all biomarkers, it has limitations. One of the main problems associated with its use as a marker of brain damage is that NSE concentrations could be affected by hemolysis. Erythrocytes contain a large amount of NSE; hemolysis may, therefore, cause a marked increase of NSE in the blood. Furthermore, an increase in NSE has been documented in patients with multiple traumas without head injury, and in rats with ischemic injury to abdominal organs [39]. Whether patients in the included studies experienced trauma other than head injury was rarely described, precluding any sensitivity analysis. Therefore, this aspect has not been examined and should be considered in future studies.

The strengths of this meta-analysis include the thoroughness of our systematic search, including different databases, and our comprehensive analytical approach that allowed the inclusion of studies presenting not only medians and interquartile ranges, but also means and standard deviations, thus improving the exhaustiveness of the results. Our methods all were based on guidelines for conducting and reporting systematic reviews.

Although previous narrative reviews have illustrated the potential of NSE concentrations for predicting outcome after traumatic brain injury [40]–[41], none of these used systematic review and meta-analysis methods. Extracerebral sources of NSE could lead to overestimation of the severity of the brain lesion in the early phase after TBI in patients with multiple injuries [40]. Only one study included in our meta-analysis enrolled patients without associated multiple traumas. However, the association between NSE concentrations and prognosis was consistent irrespective of other injuries. This result is concordant with observations that NSE concentrations correlate more closely with severity of brain injury than injury to any other organ. Further, serum concentrations of NSE correlate with the extent of brain damage in TBI, ischemic stroke, and intracerebral hemorrhage on computed tomography. Thus, the effect of extracerebral sources of NSE is likely to be minimal. Moreover, we could not explore the confounding effect of severity of extracerebral injuries due to lack of data.

This meta-analysis also examined the discriminative capacity of serum NSE concentrations and found only moderate discriminatory ability to predict mortality and neurological outcome in TBI patients. However, so far the resulting prognostic value of NSE remains low in spite of observed associations. Given the lack of information on TBI patients’ outcomes that trauma medical teams and neurosurgeons faced with decisions about level of care currently have, even a moderately reliable indicator may be useful. Serum NSE could be combined with other predictors such as Glasgow coma scale, age, pupillary reaction, head CT, and data from electrophysiological tests to develop a prognostic model to optimize level of care. However, the relatively higher negative predictive value of NSE to exclude a clinically important brain injury could be useful to determine whether to perform additional diagnostic assessments such as CT scans in TBI patients, thus avoiding exposure to unnecessary radiation, allowing better use of resources, and controlling costs.

Many questions remain unanswered, such as the prognostic cutoff value and the optimal assay and time of sampling, which might affect the cutoff value. With the current level of evidence, we could not determine serum NSE values that distinguish prognoses. Further research is needed to develop a prognostic model with high discriminative capacity based on a combination of variables known to be associated with TBI outcomes.

### Conclusions

In this systematic review and meta-analysis of the prognostic value of serum NSE in traumatic brain injury patients, we found that unfavorable outcome and mortality were significantly associated with greater NSE concentrations. In addition, serum NSE has moderate discriminatory ability to predict mortality and neurological outcome. The optimal discrimination cutoff values for NSE and the optimal sampling time remain uncertain, as there were important variations between studies. Further efforts should focus on standardizing assay, identifying optimal cutoff values and sampling time, and on combining NSE concentrations with other prognostic indicators to improve the accuracy of prognostic models to guide level-of-care decisions.

## Supporting Information

Checklist S1 **PRISMA Checklist.**
(DOC)
